# Greenhouse gas emissions from agricultural food production to supply Indian diets: Implications for climate change mitigation

**DOI:** 10.1016/j.agee.2016.12.024

**Published:** 2017-01-16

**Authors:** Sylvia H. Vetter, Tek B. Sapkota, Jon Hillier, Clare M. Stirling, Jennie I. Macdiarmid, Lukasz Aleksandrowicz, Rosemary Green, Edward J.M. Joy, Alan D. Dangour, Pete Smith

**Affiliations:** a Institute of Biological and Environmental Sciences, University of Aberdeen, Aberdeen AB24 3UU, UK; b International Maize and Wheat Improvement Centre (CIMMYT), Sustainable Intensification Program, NASC Complex, New Delhi 110012, India; c International Maize and Wheat Improvement Centre (CIMMYT), Sustainable Intensification Program, Apdo, Postal 6-641, 06600 Mexico, Distrito Federal, Mexico; d Rowett Institute of Nutrition and Health, University of Aberdeen, Aberdeen AB25 2ZD, UK; e Faculty of Epidemiology and Population Health, London School of Hygiene & Tropical Medicine, Keppel Street, London WC1E 7HT, UK; f Leverhulme Centre for Integrative Research on Agriculture and Health, London WC1H 0PD, UK

**Keywords:** Agriculture, Cool Farm Tool, Greenhouse gas emissions, Indian diets, Sustainability

## Abstract

Agriculture is a major source of greenhouse gas (GHG) emissions globally. The growing global population is putting pressure on agricultural production systems that aim to secure food production while minimising GHG emissions. In this study, the GHG emissions associated with the production of major food commodities in India are calculated using the Cool Farm Tool. GHG emissions, based on farm management for major crops (including cereals like wheat and rice, pulses, potatoes, fruits and vegetables) and livestock-based products (milk, eggs, chicken and mutton meat), are quantified and compared. Livestock and rice production were found to be the main sources of GHG emissions in Indian agriculture with a country average of 5.65 kg CO_2_eq kg^−1^ rice, 45.54 kg CO_2_eq kg^−1^ mutton meat and 2.4 kg CO_2_eq kg^−1^ milk. Production of cereals (except rice), fruits and vegetables in India emits comparatively less GHGs with <1 kg CO_2_eq kg^−1^ product. These findings suggest that a shift towards dietary patterns with greater consumption of animal source foods could greatly increase GHG emissions from Indian agriculture. A range of mitigation options are available that could reduce emissions from current levels and may be compatible with increased future food production and consumption demands in India.

## Highlights

• Highest GHG emissions from food production are from rice and ruminant products.
• Highest GHG emissions from consumption are from rice and livestock products.
• Consumption choice can either increase or decrease total GHG emissions.

## 1 Introduction

Agriculture is an important sector of the economy in India, contributing about 20% of national gross domestic product, and providing a livelihood for nearly two-thirds of the population (ICAR, 2015). Equally important is the contribution of agriculture to national food security. India achieved self-sufficiency in food production after the Green Revolution (GR), but retaining this success has been challenging due to the increasing scarcity of resources, including labour, water, energy, and rising costs of production (Saharawat et al., 2010). Increased use of production inputs, such as mineral fertiliser, has made Indian agriculture more greenhouse gas (GHG)-intensive. Agricultural production is a major emitter of GHGs, currently accounting for 18% of total GHG emissions in India (INCCA, 2010). Recent estimates report that global food production must increase by 70% to meet the projected food demand of the estimated 9 billion global population by 2050 (CTA-CCAFS, 2011). With a population of ∼1.3 billion, it is evident that the food system in India will be central to the global challenge of providing sufficient nutritious food while minimising GHG emissions. However, given the increasing population and shifting dietary patterns, GHG emissions from agricultural production in India are expected to change.

Quantifying GHG emissions associated with the production of food items in India is an important stage in quantifying GHG emissions associated with diets. It allows us to (i) identify variation in GHG emissions between typical dietary patterns within India; (ii) forecast the effect of changes in diets on GHG emissions; and (iii) identify options to minimise GHG emissions from food production, either through production-side changes or through dietary changes. For example, a number of countries have experienced a ‘nutrition transition’ associated with greater disposable incomes, urbanisation and globalisation. The transition is typified by increasing consumption of animal products, edible oils and sweetened beverages and decreasing consumption of cereals and pulses (Drewnowski and Popkin, 1997, Popkin et al., 2012). There is evidence that a similar trend is emerging among some population groups in India, although cultural preferences for lacto-ovo-vegetarian diets suggest that India’s experience will differ from other countries including China (Baker and Friel, 2014, Misra et al., 2011). The implications of dietary changes in India for GHG emissions have not been quantified.

In India, the majority of agricultural GHG emissions occur at the primary production stage (Pathak et al., 2010), and are generated through the production and use of agricultural inputs, farm machinery, soil disturbance, residue management and irrigation. These practices are used to increase yields and improve harvests. Due to its direct contribution to global GHG emissions, agriculture can also serve as an important climate change mitigation strategy (Smith et al., 2013, Smith et al., 2008), both by reducing GHG emissions to the atmosphere, and by sequestering atmospheric carbon into plant biomass and soil, though the role of some soil carbon sequestration practices for climate mitigation has been questioned (Powlson et al., 2014). India’s Intended Nationally Determined Contributions (INDCs) to the United Nations Framework Convention on Climate Change (UNFCCC, http://unfccc.int/2860.php [accessed 19.05.2016]) place emphasis on mitigation from agriculture, and various mitigation strategies (particularly concerning methane, CH_4_, and nitrous oxide, N_2_O) have been proposed (Smith et al., 2014, Smith et al., 2008). Quantification of GHG emissions from the production of different food commodities helps farmers, researchers and policymakers to understand and manage these emissions, and identify mitigation responses that are consistent with the food security and economic development priorities of countries (Hillier et al., 2011, Whittaker et al., 2013).

Various methods exist to estimate GHGs from agriculture, ranging from simple Tier 1 methods (IPCC 2006) to complex process-based models, which simulate the soil carbon and nitrogen cycles in some detail (Ogle et al., 2013). Several tools and calculators have been developed for estimating GHG fluxes from farm activities and to support decision making in terms of identifying informed interventions. Here, we used a modified version of The Cool Farm Tool (Hillier et al., 2011), which integrates several empirical models into one tool for GHG estimation from farm activities. The tool recognises context-specific factors that influence GHG emissions such as pedo-climatic characteristics, production inputs, and other management practices at farm level. GHG emissions from livestock products are calculated using the comprehensive data from the 19th Livestock Census of the Government of India (GOI, 2012) following the approach of Herrero et al. (2013).

The objective of the study is to analyse and compare farm-level GHG emissions of major food commodities at a national scale in India. The study gives an overview of emission-related hotspots, and discusses the implications for low carbon development in relation to changing diets in India.

## 2 Materials and methods

### 2.1 Food items

We calculated GHG emissions from agricultural production of several crops and livestock products in India (Table 1). Items for analysis were chosen from the total list of consumed foods recorded in the Indian Migration Study (IMS), a regional survey that measured dietary intake in 2005–2007 (Bowen et al., 2011). Based on project logistics, we set an objective of analysing a set of 20 food items. We used two criteria to choose items for analysis: having at least one item from each broad food group (i.e., cereals, pulses, tubers, vegetables, etc.); and within each group, selecting items reported to be consumed in the greatest quantity within the study (in kg *capita*^−1^ d^−1^). In total, 17 single food items were analysed, including 13 single crops, and four animal-sourced products (Table 1), plus three crop groups (other cereals, other pulses and crops used for vegetable oils). These items represent about 72% of reported consumption of food (kg) in the IMS, and about 75% of consumption when assessed in a 2012 nationally-representative household expenditure survey (National Sample Survey Organisation, 2007). The livestock products included in the analysis are milk, eggs, poultry and mutton meat. Fish and seafood were excluded from the study. This is because the consumption of these groups was low within the IMS sample which meant that the additional methodological effort and data acquisition required to conduct the analysis was not justified. Our study therefore focusses on agricultural produce.

### 2.2 Management data

**Fig. 1:**
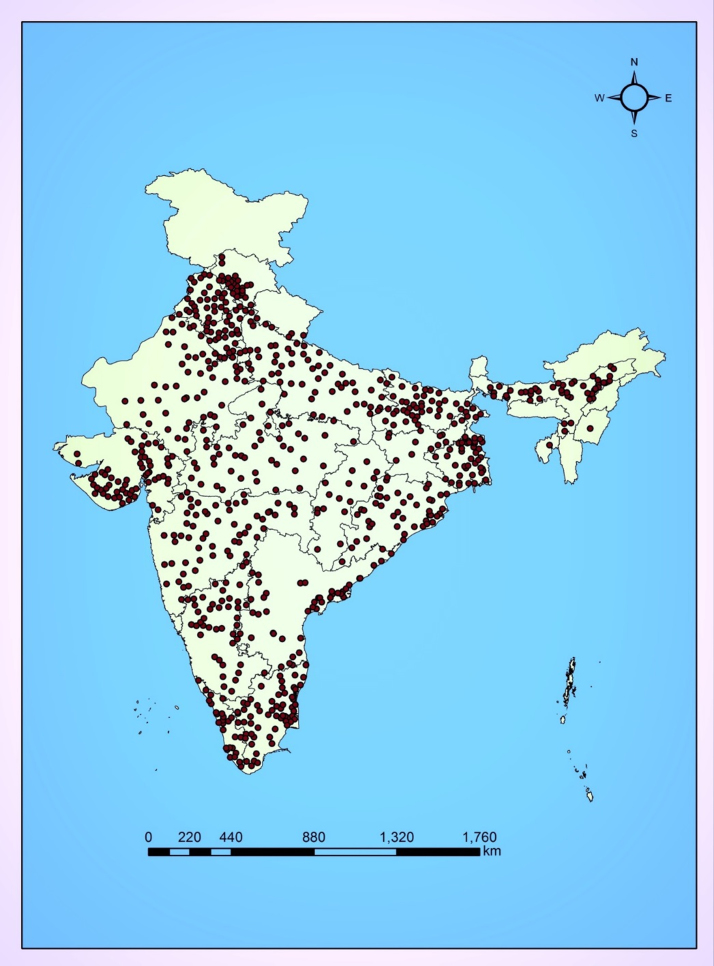
Location of sampled villages for the cost of production survey in India, from which activity data were derived for this study.

Agricultural input and management information, including yield of major crops grown in India, were obtained from the Directorate of Economics and Statistics of the Government of India (http://eands.dacnet.nic.in [accessed 01.10.2015]). The Government of India conducts cost of cultivation surveys at the Indian district level using multi-stage sampling. Districts within states, and villages within districts, formed the first and second stage unit of sampling with the ultimate unit of data collection being the household (CSO, 2002). The district and villages were selected in order to cover the major crops grown in the country. Fig. 1 shows the locations of households selected for the survey, which forms the foundation of the activity data used in this study. In total, there were 34,577 data points across India used in the study. Of these, 53% of data points were for paddy rice and wheat, representing the proportionate area under rice and wheat cultivation in India. Data on temperature and rainfall were obtained from the WorldClim global climate database (http://worldclim.org/ [accessed 01.10.2015]), and soil data (soil texture, soil organic carbon, soil pH, bulk density) were obtained from Shangguan et al. (2014). The water management system before and during rice cultivation was determined from databases at national and state levels (Gupta et al., 2009), and expert opinion (experts from CIMMYT). The analysis includes a representative distribution of irrigation management strategies for rice, from flooded to alternative wetting and drying systems. In India, agricultural residues left in the field after harvest are sometimes burnt *in-situ* to facilitate cultivation of subsequent crops, or used for other purposes off-site. The information on residue management of different crops, including burning, was obtained from Gadde et al. (2009) and Jain et al. (2014) at state level. The area under different crop cultivation in each state and union territory were obtained from state agriculture departments, the Directorate of Economics and Statistics of the Government of India, and FAOSTAT (FAOSTAT, 2015).

State-wise details of livestock by breed, age, sex and management type were obtained from the 19th Livestock Census of the Government of India (GOI, 2012). The information on livestock body weight, feed consumption and *per-capita* production of meat and milk (Table 2) were based on Singhal and Mohini (2002) and on expert judgement from the National Dairy Research Institute (NDRI) following relationships outlined in Herrero et al. (2013).

Management information for crops not included in the data set from the Directorate of Economics and Statistics of the Government of India, was generated from another source of general management information (http://www.haifa-group.com/knowledge_center/recommendations/fruit_trees/ [accessed 01.06.2015]), and statistics from FAOSTAT (2015).

### 2.3 Model and greenhouse gas emissions

GHG emissions from crops were calculated using the Cool Farm Tool (CFT) (Hillier et al., 2011; CFT: https://www.coolfarmtool.org/ [accessed 01.10.2015]). The CFT is a GHG emission calculator which allows users to estimate annual GHG emissions associated with the production of crops or livestock products from production to the farm gate (Hillier et al., 2011). It comprises a generic set of empirical models that are used to estimate full farm-gate product emissions constituting a mix of Tier 1, Tier 2, and simple Tier 3 approaches (see IPCC, 1997 for definitions of tiers for GHG estimation in national GHG inventories). GHG emissions were estimated from inputs including general information about soil and climate, and the set of management options on the farm: fertilisation, pesticide and herbicide use, residue management, machinery and energy use.

For the current analysis, a version of the CFT implemented in Matlab (R2012a [7.14.0739], MathWorks, USA) was used to calculate the emissions for on-farm plots across India. The exception was for rice production where the method of Yan et al. (2005) was preferred to the Cool Farm Tool (which uses the Tier 1 method of IPCC (2006)), due to the greater granularity of the Yan et al. method, which bases estimates of CH_4_ emissions on several variables (i.e. soil pH, climate, organic amendment, pre-water regime, water regime) which were available at plot level in this study but were not factored in to the IPCC tier 1 method (IPCC, 2006).

GHG emissions from livestock products were calculated using the approach of Herrero et al. (2013) which provides data on GHG emissions from enteric fermentation and manure management for several animal groups (i.e. ruminants, small ruminants, pigs and poultry) using data for India on livestock systems and feed. National GHG emissions were calculated based on the average body weight of the livestock for different regions. Additional emissions for feed production were calculated using the CFT for feed crops.

We account only for GHG emissions related to farm management, and do not account for processing or transport after the farm-gate. GHG emissions up to the farm gate are reported in CO_2_ equivalent (CO_2_eq) *per* ha of crops and *per* head for livestock using the 100 year global warming potentials used in national GHG accounting (IPCC, 2006). For comparison, all results are also presented on a *per* kg production basis. GHG emissions were also converted to kg CO_2_eq kcal^−1^ using FAO (2001) data for the energy content of the food commodities.

## 3 Results

### 3.1 GHG emissions of food items up to the farm-gate

**Fig. 2:**
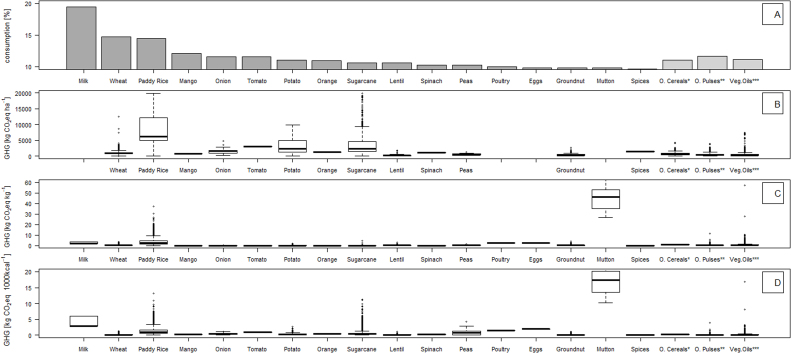
(A) mass of food group consumption as a%-age of total consumption reported in the Indian Migration Survey (reference); (B) GHG emissions associated with production of food groups in India per hectare, (C) per kg yield, (D) per 1000 kcal. (*Other Cereals: includes bajra, barley, maize, ragi and jowar; ** Other Pulses: includes black, red and green gram; *** Crops for vegetable oils: includes coconut, rapeseed, soybean, safflower, sesamum, sunflower).

The analysis of GHG emissions from farm management in India presents the variability of emissions across India based on different management practices (Fig. 2, Table 1). There are more data for the most widely consumed crops (i.e. wheat and rice; Table 1) than other products. The variability in GHG emissions for wheat is less than that for rice or potato. For paddy rice, >10,000 plots were available for analysis with a wide range of management practices, which is reflected in the GHG emission results. The main reason for the wide range in GHG emissions seen in rice is water management, which is the main determinant of CH_4_ emissions. In particular, continuous flooding generates the highest CH_4_ emissions, while longer and more frequent periods of water drainage reduces emissions. For example, changing the water regime from continuously flooded to multiple drainage periods, reduces CH_4_ emissions by 9-fold (data not shown). High emissions on a *per*-ha-basis correspond with high GHG emissions *per* kg rice.

With the exception of flooded rice, the major source of variation in GHG emissions for crops is due to variation in fertiliser application. The results show a wide range of GHG emissions for rice, potato and sugarcane on a *per*-ha-basis, and a narrow range for other crops (Fig. 2B). The groups “other cereals” (i.e. bajra, barley, maize, ragi and jowar), and “other pulses” (i.e. black, red and green gram), had broadly similar GHG emissions. Emissions from vegetable oil crops showed more variation across the different crops (i.e. coconut, rapeseed, soybean, safflower, sesamum, sunflower).

GHG emissions *per* kg of livestock product (Fig. 2C) varies markedly between livestock types. GHG emissions are highest for mutton meat (as the example for ruminant meat), followed by other livestock production such as poultry and dairy (milk). GHG emissions *per* kg of product were greater for livestock products than for crops, with the exception of rice. Mean GHG emissions were <1 kg CO_2_eq kg^−1^ product for all crops except rice, with decreasing emissions across the categories of spices, pulses and nuts, wheat, fruits, vegetables and roots, and sugarcane, respectively (Table 1).

GHG emissions *per* kcal show a different ranking, although products from ruminant animals have the highest emissions using all metrics. GHG emissions *per* kcal show a small increase across cereals, pulses, vegetables, fruits and animal-source foods. Rice in particular had higher emissions than other crops, and mutton meat had markedly high emissions, several times more than other animal-source products.

### 3.2 GHG emissions from food consumption in India

**Fig. 3:**
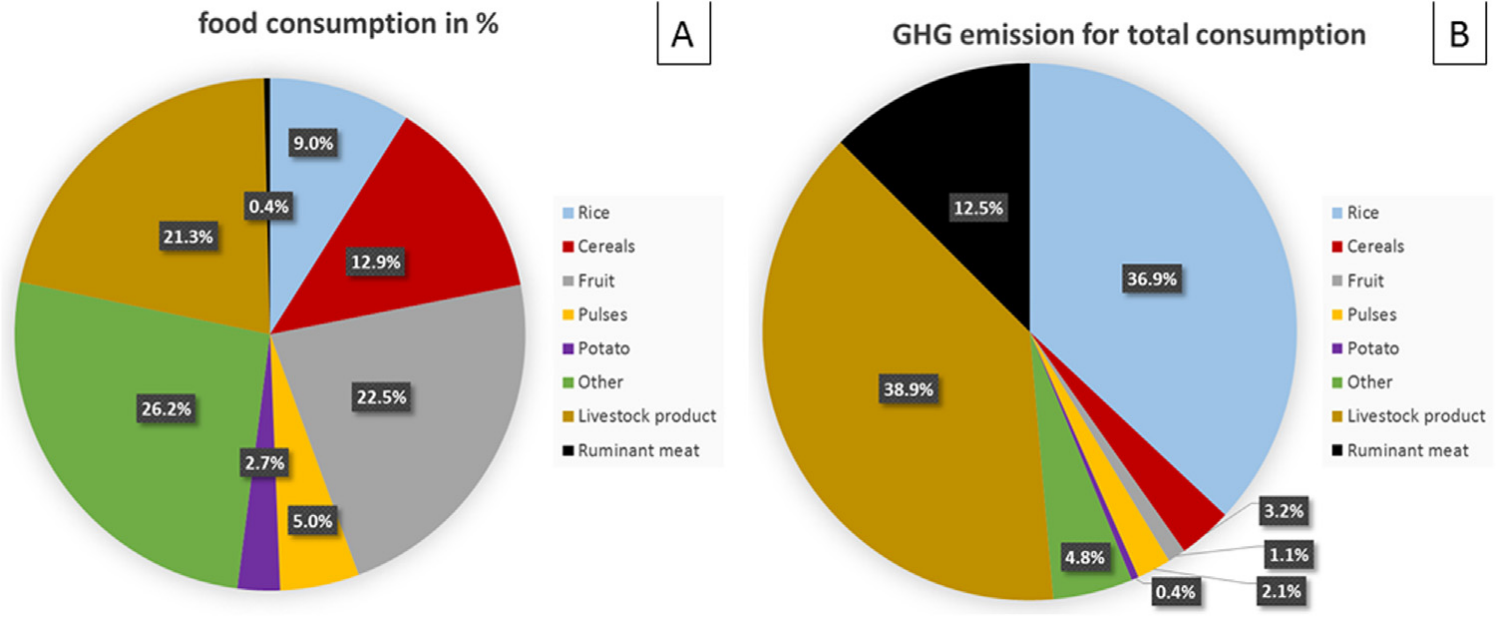
Proportion of consumption of food groups in Indian diets (A), and distribution of GHG emissions from agricultural production of this diet (B).

Fig. 3A shows relative reported consumption by weight of commodities in the IMS, while 3B shows their relative contribution to emissions. Rice and livestock products contribute the most to total dietary GHG emissions, with the third contributor being ruminant meat. Although ruminant meat had the greatest GHG emissions *per* unit product, it contributed less to overall GHG emissions (12.5%) as consumption is low, accounting for only 0.4% of total intake. Cereals other than rice and fruit products account for 12.9% and 22.5% of reported consumption by weight, yet as their emissions *per* unit of product are low, they make a relatively small contribution to total dietary GHG emissions, representing only 3.2% and 1.1% of total emissions, respectively. The group “other” (including various crops from the subgroups nuts and oils, spices, and vegetables) also contributed little to total dietary GHG emissions.

## 4 Discussion

### 4.1 GHG emissions from crop production

GHG emission calculations for agricultural production of the commonly consumed food items in India are based on a substantial dataset with representative plots across the country. This analysis gives an overview of GHG emissions produced through on-farm management at different locations, representing diverse soil types and climatic conditions, and encompasses major drivers of variation within the country. Using the same model to calculate GHG emissions for all of the major food groups allows emissions from different crops and products to be compared.

On a *per* ha basis, GHG emissions for major food crops in India are generally lower than those in Europe and North America, with GHG emissions for cereals 2–3-fold greater in Europe (2000–3000 kg CO_2_eq ha^−1^ yr^−1^) (Carlton et al., 2012). A study in Canada estimated GHG emissions for spring wheat of 600 and 1400 kg CO_2_eq ha^−1^ yr^−1^ (Gan et al., 2012). GHG emissions for potatoes and peas show a similar range in Europe of ∼3000 kg CO_2_eq ha^−1^ yr^−1^ and ∼660 kg CO_2_eq ha^−1^ yr^−1^, respectively (Carlton et al., 2012). A Swedish study reported GHG emissions of wheat of 0.2–0.6 kg CO_2_eq kg^−1^ production (Röös et al., 2011); the calculated GHG emissions for wheat in India are towards the lower end of that range. A comparison of rice production in a Chinese study showed a range from 2000 kg CO_2_eq ha^−1^ yr^−1^ for upland rice up to 20000 kg CO_2_eq ha^−1^ yr^−1^ for paddy rice production, and similar values have been reported for Indian rice (Li et al., 2006).

The reported estimates of GHG emissions from farm management differ across the above studies partly because each uses different boundary conditions. In our study, the calculated GHG emissions on a *per*-ha-scale follow the same methods for all crops and differ mainly because of changes in management and fertiliser use for crops. For cereals in general, less fertiliser is used in India than in Europe. These differences are also partly reflected in yield. The yields for cereals, pulses and potatoes have increased over recent years in India, but are still only half of those recorded in Western Europe and North America (FAOSTAT, 2015). These differences show the importance of comparing GHG emissions on a *per*-kg-production-basis, as GHG emissions will be greater for low yielding crops than for higher yielding ones using a *per*-kg metric. For instance, according to FAOSTAT (2015) rice yields in China are around twice those in India.

The highest GHG emissions among crops are associated with paddy rice production. Emissions of CH_4_ from rice production are recognised as a significant source of GHG emissions globally, and many studies show that changes in water management can substantially reduce CH_4_ emissions (Liu et al., 2010, Nayak et al., 2015, Yan et al., 2005). It is possible to reduce CH_4_ emissions and increase yield through optimising drainage and manure management (Banerjee et al., 2002, Malla et al., 2005, Thu et al., 2016). Specifically, changing a continuously-flooded system to intermittent irrigation shows potential to greatly reduce CH_4_ emissions. Although some studies show that N_2_O emissions may increase under intermittent irrigation the decrease in CH_4_ emissions more than compensates this effect (Liu et al., 2010, Nayak et al., 2015). Nayak et al. (2015) summarised management opportunities to mitigate GHG emissions from agriculture in China, and these can largely be adapted to Indian agriculture. In rice management, key elements are fertiliser management by reducing synthetic fertiliser inputs, increasing organic manure, and improved water management, as discussed above.

### 4.2 GHG emissions from livestock products

As expected, GHG emissions from livestock products are generally higher than those from crop production (Fig. 2, Table 1). This reflects the inefficiencies of conversion of plant protein to animal protein in herbivores (Ripple et al., 2014), and is also impacted by additional sources of emissions resulting from manure management and enteric fermentation in ruminants. GHG emissions associated with livestock products depend largely on feed inputs, and in other studies have been shown to range between 0.8–2.4 kg CO_2_eq kg^−1^ milk, 1.7–6.6 kg CO_2_eq kg^−1^ eggs, 2.5–6.9 kg CO_2_eq kg^−1^ poultry meat and 10–20 kg CO_2_eq kg^−1^ mutton and lamb (Bellarby et al., 2013). These values are based on different studies, mainly from model exercises which focus on Europe. Our milk and poultry results for India are within the range of these studies. The calculated emissions for mutton are higher than in the above discussed studies, resulting from embedded emissions in feed, which is 50–75% of the total GHG emissions *per*-animal-*per*-year.

Ruminants produce CH_4_ through enteric fermentation, and options to mitigate this source are somewhat limited (Beauchemin et al., 2011). Other sources of emissions from livestock production are manure management and changed feed rations. To reduce GHG emissions from manure management options include (i) changes to manure storage, e.g. decreased storage time, manure storage cover with straw, or mechanical intermittent aeration during manure storage (Hristov et al., 2013), (ii) manure acidification (Ndegwa et al., 2011, Petersen et al., 2012), (iii) feeding of livestock with nitrate supplements (Van Zijderveld et al., 2011) and (iv) stacking of poultry litter (Gerber et al., 2013). To reduce GHG emissions from feed, all mitigation measures previously discussed for crops could be considered, as well as using the residues from crop production as feed.

### 4.3 GHG emissions associated with Indian diets

Overall, national GHG emissions associated with diets are greatest for rice and livestock products like milk and eggs (Fig. 3), because these are widely consumed products with high GHG emissions *per* unit of product. Although there is limited consumption of ruminant meat in India, its high GHG intensity means that it is the third greatest contributor to GHG emissions.

The mitigation potential in livestock production therefore needs to be further explored. In addition to the mitigation options in on-farm management, dietary change could help to decrease GHG emissions considerably, but advice to change dietary intakes to reduce GHG emissions would need to consider the nutritional implications, so as not to compromise health (Aleksandrowicz et al., 2016, Bajzelj et al., 2014, Smith et al., 2013, Smith, 2015). However, in the event of a nutritional transition in India, toward the consumption of a greater volume of livestock products, there is likely to be an increase in GHG emissions unless *per*-product emissions are reduced through more efficient production and targeted mitigation measures, especially in the livestock sector and for rice production.

## 5 Conclusion

This study constructed a national dataset of GHG emissions associated with the production of major food items in India, and incorporates variability in emissions from a range of production systems. We used comprehensive agricultural activity data at the farm-level, and a state-of-the-art greenhouse gas accounting tool. We highlight the risk of a likely increase in GHG emissions if diets transition towards increased consumption of animal-based products, and also observe a wide range of emissions from cereal production. In addition to general measures to improve efficient use of nutrients and organic matter stocks, there is also likely to be benefit in developing support mechanisms to target those products with the highest emissions *per* unit of production. We hypothesise that in such cases, mitigation of GHG emissions will be a co-benefit of improved and more efficient agronomic practice, but these options would have to consider the nutritional and health implication for the Indian diet.

## Figures and Tables

**Table 1:** Major crops and livestock products by % of total intake in India, number of data points available with management information, averaged data and standard deviation for each product, nitrogen input and GHG emissions for different scales.

crop/livestock prod.	group	subgroup	% of consumption from total food in Indian diets	nr. of data points	yield [tonnes/ha]	std dev	N [kg/ha]	std dev	GHG [kg ha^−1^]	std dev	GHG [kg kg^−1^ product]	std dev	GHG [kg 1000 kcal^−1^]	std dev
Milk	Livestock product	Dairy-lo-fat	18.17	105							2.42	0.90	3.97	1.48
Wheat	Cereals	Cereals	9.42	6017	3.26	1.14	139.41	51.47	977.15	439.70	0.34	0.21	0.12	0.07
Paddy Rice	Rice	Cereals	8.97	11993	3.61	1.51	114.37	54.21	8447.59	4754.41	5.65	4.59	1.21	0.96
Mango^a^	Fruit	Fruit	4.60	/	10.4	/	11.7	/	750.00		0.07		0.16	
Onion	Other	Spices	3.72	48	19.55	8.59	192.57	98.24	1599.65	969.44	0.10	0.07	0.39	0.29
Tomato^a^	Other	Vegetable	3.67	/	130	/	360	/	3000.00		0.15		0.88	
Potato	Potato	Tuber	2.69	394	23.83	9.27	236.01	181.24	3406.46	2727.19	0.22	0.23	0.33	0.35
Orange^a^	Fruit	Fruit	2.57	/	10.3	/	113	/	1300.00		0.13		0.37	
Sugarcane	Other	Other	1.90	1312	79.35	33.49	258.84	122.67	3954.34	3975.21	0.09	0.22	0.73	2.07
Lentil	Pulses	Pulses	1.89	425	0.90	0.39	16.03	14.96	292.17	303.45	0.38	0.38	0.13	0.13
Spinach	Other	Vegetable	1.29	/	21	/	33.5	/	1100.00		0.05		0.30	
Peas	Pulses	Pulses	1.17	128	1.39	0.75	41.41	38.11	540.09	250.37	0.42	0.21	0.81	0.84
Poultry	Livestock product	Chicken	0.74	69							2.59	0.08	1.40	0.04
Egg	Livestock product	Egg	0.45	69							2.59	0.08	1.87	0.06
Groundnut	Pulses	Nuts and oils	0.39	629	1.36	0.73	50.66	44.71	383.58	295.60	0.38	0.47	0.10	0.13
Mutton	Ruminant meat	Meat	0.38	280							45.54	11.89	17.32	4.52
Spices (Cumin Seed)	Other	Nuts and oils	0.08	/	2	/	100	/	1500.00		0.75		0.25	
Other Cereals^b^	Cereals	Cereals	2.76	3520	1.94	1.41	64.43	53.65	707.32	377.03	0.43	0.50	0.06	0.13
Other Pulses^c^	Pulses	Pulses	3.8	3720	0.82	0.57	25.14	36.50	490.32	359.31	0.75	1.59	0.14	0.38
Crops for vegetable oils^d^	Other	Nuts and oils	2.84	2569	1.30	0.62	40.66	36.20	532.12	632.39	0.54	0.93	0.12	0.18

a No plot/farm data were available; typical management and statistical information were used to generate management information.

b Includes bajra, barley, maize, ragi and jowar.

c Includes black, red and green gram.

d Includes coconut, rapeseed, soybean, safflower, sesamum, sunflower.

**Table 2:** Livestock body weight, feed consumption and per-capita production of meat and milk used in the analysis.

						Feed type		
livestock	breed	age/category	body wt (kg)	product (meat/milk)	meat (kg/animal/year)	dry fodder (kg/animal/year)	green fodder (kg/animal/year)	concentrates (kg/animal/year)
sheep/mutton	exotic	under 1 year	24	meat	14	365	1460	109.5
sheep/mutton	local	under 1 year	18	meat	10	365	1277.5	116.8
sheep/mutton	exotic	more than 1 year	42	meat	23	547.5	2190	146
sheep/mutton	local	more than 1 year	38	meat	20	365	1642.5	109.5
cattle	exotic	milk	350	milk	2555	1460	8030	730
cattle	local	milk	300	milk	1095	1460	6570	730
poultry			1.8	meat	10 kg/cycle			30 kg/cycle
poultry			1.5	egg	315 egg/cycle			82 kg/cycle
